# Adolescents and Young Adults Evaluating a Website for Affective-Sexual Information and Education: A Mixed-Methods Study Protocol

**DOI:** 10.3390/ijerph192416586

**Published:** 2022-12-09

**Authors:** Laura Montero-Pons, Gemma Falguera-Puig, Rosa García-Sierra, Josep Maria Manresa-Domínguez, Azahara Reyes-Lacalle, Rosa Cabedo-Ferreiro, Judit Cos-Busquets, Ana Zaragoza Marfà, Maria Pilar Sancho Pérez, Dolors Rodríguez-Martín

**Affiliations:** 1 Doctoral Program in Nursing and Health, Faculty of Medicine and Health Sciences, School of Nursing, Universitat de Barcelona, 08907 L’Hospitalet de Llobregat, Spain; 2 Sexual and Reproductive Healthcare Santa Coloma de Gramenet, Primary Care Management Metropolitana Nord, Catalan Institute of Health, 08921 Santa Coloma de Gramenet, Spain; 3 Research Group on Sexual and Reproductive Healthcare (GRASSIR) (2017-SGR-793), 08007 Barcelona, Spain; 4 Coordinator of Sexual and Reproductive Healthcare Metropolitana Nord, Primary Care Management Metropolitana Nord, Catalan Institute of Health, 08203 Sabadell, Spain; 5 Multidisciplinary Research Group in Health and Society (GREMSAS) (2017-SGR-917), 08007 Barcelona, Spain; 6 Research Support Unit Metropolitana Nord, Primary Care Research Institut Universitari d’Investigació en Atenció Primària Jordi Gol (IDIAP JGol), 08303 Mataró, Spain; 7 Nursing Department, Faculty of Medicine, Campus Bellaterra, Universitat Autònoma de Barcelona, 08193 Barcelona, Spain; 8 Primary Care Group, Germans Trias i Pujol Research Institute (IGTP), 08916 Badalona, Spain; 9 Sexual and Reproductive Healthcare Sabadell, Primary Care Management Metropolitana Nord, Catalan Institute of Health, 08203 Sabadell, Spain; 10 Sexual and Reproductive Healthcare Granollers, Primary Care Management Metropolitana Nord, Catalan Institute of Health, 08400 Granollers, Spain; 11 Coordinator of Sexual and Reproductive Healthcare Muntanya, Primary Care Management Barcelona Ciutat, Catalan Institute of Health, 08030 Barcelona, Spain; 12 Department of Fundamental Care and Medical Surgital Nursing, Faculty of Medicine and Health Sciences, School of Nursing, Universitat de Barcelona, 08907 L’Hospitalet de Llobregat, Spain; 13 Consolidated Research Group on Gender, Identity and Diversity (2017-SGR-1091), 08007 Barcelona, Spain

**Keywords:** sex education, adolescent, young adult, internet, focus groups, qualitative research, mixed methodology

## Abstract

The website Sexe Joves is a website on sexuality of the Department of Health of the Government of Catalonia (Spain). This study aims to understand the experiences and opinions of people aged 14 to 25 regarding this website, taking into account sex, gender identity, sexual orientation, socioeconomic status and location within Catalonia (urban, semiurban and rural areas). With the objective of improving the website and adpating the resources allocated to it, this study evaluates whether this population is familiar with it and uses it, as well as the website’s usability and accessibility (digital equity), usefulness and the relevance of its content. A parallel convergent triangulation design is used: a qualitative study using a social constructivist perspective, and an observational, descriptive and cross-sectional quantitative study. We conduct a discourse analysis of participants and use an “ad hoc” questionnaire to collect quantitative data. A descriptive analysis of all variables is carried out. Affective-sexual education aimed at young people must stem from their participation and the whole range of sexual and gender diversity in order to reach the entire population equally. This analysis will contribute to the design of new strategies for the wesbite Sexe Joves, a public health resource, in order to improve affective-sexual education for young people.

## 1. Introduction

Health professionals have implemented sex education for adolescents for decades. Oftentimes, such education has not been based on a holistic model of health but focused solely on preventing sexually transmitted infections (STIs) and unplanned pregnancies. This model replicates heteronormative sexuality and drives young people to use mainstream pornography [1].

Affective-sexual education for young people pivots on a positive sexuality, which is understood as something to be enjoyed, not centred around fear, and removed from the coitocentric, biomedical, heteropatriarchal and binary model. This type of sexuality is based on self-esteem and self-care, as well as respect and empathy [1]. It should be a dynamic and holistic process that includes sharing information, enabling communication, and the transformation of attitudes, beliefs and values. Broadly, the objectives are young people taking responsibility for their own actions and behaviours, equality between the different sexes and gender identities, acceptance, intolerance of violence, and empathy. In this way, affective-sexual education should represent a new paradigm that consists of working with adolescents and young adults on affection, emotions, interpersonal relationships and pleasure [1,2] so that they can fully enjoy a healthy sexuality that respects themselves and others, from a comprehensive biological, psychological and social perspective.

Global data support the need for this type of research. Violence against women by their intimate partners and others is currently considered a major public health problem [3]. In Spain, the number of complaints against minors due to gender violence tripled between 2008 and 2017 [4]. Unfortunately, sexist attitudes within couples persist in younger generations. An astonishing 27% of young people in Spain believe that gender violence is normal within couples, and over 80% reported being aware of acts of abuse in couples in their age range [4]. Data on sexual assault not perpetrated by intimate partners from 15 years of age show that between 0.3% and 12% of women have suffered some act of sexual violence [5]. Furthermore, studies have shown that the highest risk of sexual violence occurs during adolescence and young adulthood [6].

Statistics show an upward trend in the diagnosis of STIs, a consequence of risky sexual practices without the use of barrier methods [7]. Worryingly, it appears that only a minority of young people in Europe have adequate information on sexual issues [8]. Experts emphasize the need to improve sexual education, start education on gender roles from an early age and address the harmful effects of the romantic love paradigm [9]. They also underscore that we cannot just treat STIs, but need to use a personalized psychosocial approach for each case [10,11].

In agreement with the Istanbul Convention [12], discussions on gender equality should be urgently implemented at all levels of education. Affective-sexual education is an essential element of the 2030 Agenda for Sustainable Development and its 17 Sustainable Development Goals (SDGs) approved by world leaders in 2015 [13].

In 2016, the Guttmacher Institute and The Lancet created a commission on Sexual and Reproductive Health and Rights (SRHR). They concluded that the 17 SDGs are insufficient for a broad implementation of SRHRs [14]. In their report, they denounce inequities between and within countries where a sector of the population cannot access adequate resources. The commission underscored the need for a comprehensive look at sexual and reproductive health, with special emphasis on adolescent sexuality, gender-based violence, abortion and the diversity of sexual orientations and gender identities [15]. Additionally, the socioeconomic perspective indicates that women in the lower socioeconomic strata use less effective contraception and are at higher risk of unplanned pregnancies [16]. Studies indicate that inequalities in sexual and reproductive health occur both in migrants and native residents of disadvantaged urban neighbourhoods [17].

In order to improve these indicators, guidelines and programs on sexual health for young people should be re-evaluated and include the thoughts, experiences and opinions of the target population. The result should be an affective-sexual education that is truly effective.

### 1.1. Background

Affective-sexual health education for young people in Catalonia is provided by the public universal primary care system, specifically from the Reproductive and Sexual Healthcare units (ASSIR, Atenció a la Salut Sexual i Reproductiva) [2,18]. This education can be imparted during individual consultations, youth community events (for instance, the Youth Afternoon), and also from institutional websites [19].

The website Sexe Joves (WSJ) on sexuality is a reference for health professionals in Catalonia (www.sexejoves.gencat.cat). The project started in 2003 and was fully operative in 2006. It was led by midwives from the outset, with support from the government of Catalonia and the Catalan Institute of Health (Institut Català de la Salut (ICS)). A multidisciplinary team of 65 professionals from various institutions related to sexuality and youth participated in the creation process. This group prepared a document with content for the website, which was validated by a total of 153 young people from secondary schools from across Catalonia [19]. The main aims are twofold: (1) to help young people enjoy a healthy and responsible sexuality; and (2) to prevent unplanned pregnancies and STIs. The website offers information on affection, self-esteem, knowledge about one’s body, abuse, harassment, sexual assault, sex and drugs, and virtual sex. It also includes a virtual consultation room (email and chats), where a team of midwives provides individualized care, and links to health centres that offer face-to-face and/or telephone consultations.

The website is designed for 14- to 25-year-olds and is accessible to people with hearing, eye and motor impairment. It is also used as a consultation tool for adults, health professionals, educators and others. The advice takes into account sex, sexual orientation and gender identity [20]. The WSJ has a multidisciplinary editorial committee, which consists of nine midwives, two psychologists, a gynaecologist, an administrator and two senior public health technicians from the Catalan government, and is coordinated by the midwife who launched the project. The committee is responsible for the day-to-day running of the website, updating its content and analysing indicators such as number of visits, telematic consultations and most consulted topics [20].

Although the website has been operative for 16 years, it was only evaluated in 2012, as part of a doctoral dissertation [19]. Through a quantitative study with a descriptive design that analysed sevaral variables from the website, as well as the user satisfaction of young people, it was concluded that at that time, the WSJ had a high usability (in a website or computer program refers to ease of use, taking into account readability of texts, speed of download, manageability and capacity to meet the needs of the user [21]) rate. In the second phase of the same doctoral dissertation, an analysis was conducted to evaluate the quality of responses from the midwives with a validated questionnaire. In the third phase, a quasi-experimental study was performed, pre- and post-training, of the midwives in charge of responding to the website’s virtual consultation. The dissertation conluded that the quality of responses to emails was high and that up-to-date training of professionals who answer these emails would be sufficient to reduce inadequate responses [19]. The editorial committee has now commissioned the research team of this project to undertake an in-depth evaluation of the website centred around the voice of the youth of Catalonia.

The Sexunzipped website for the UK population over the age of 16 is one of several affective-sexual education websites that have been created and/or evaluated through research projects [22,23]. Bailey’s group has used this website for quantitative and qualitative research projects. In 2012, they conducted a qualitative study with people aged 16 to 22 that included 21 focus groups and 6 in-depth interviews to understand preferences regarding the content and format of Sexunzipped [24]. They later published a randomized trial describing the quantitative evaluation of the website and its content [25]. In 2013, they presented the results of a mixed-methodology study that used in-depth interviews and analysis of emails/chats and questionnaires to explore the views of young people on STI screening using Sexunzipped and mail-in samples [26]. In December 2013, Bailey’s group used Sexunzipped to conduct an online randomized controlled trial with people aged 16 to 20 living in the United Kingdom to assess the feasibility of the various dimensions of the design of online clinical trials [27]. In the last five years, Bailey’s group has evaluated interventions using mobile text messages, tablets in the waiting rooms of sexual health services, and social networks [28,29].

Templeton et al. also investigated the promotion of sexual health in young people, in their case using a qualitative methodology with participatory interventions and prison populations. In their systematic review, they conclude that qualitative and participatory methodologies that incorporate what young people really want are essential for effective change [30].

The literature review supports the use of mixed methods to explore the point of view of young people on the WSJ. The results of this study will contribute essential data to determine new strategies for the sustainability and relevance of the WSJ. It also aims to provide a holistic perspective of affective-sexual health that includes (1) sexual and gender diversity and (2) socioeconomic factors. The results might also provide advice for professionals of primary healthcare, school health and community nursing.

### 1.2. Aims

#### 1.2.1. General Objective of the Project

To evaluate the WSJ as a source of affective-sexual health information, education and communication for young people.

#### 1.2.2. Specific Objectives of the Project

- To outline a new participatory model of affective-sexual education for adolescents and young adults led by midwives and/or nurses that incorporates the gender perspective and sexual and socioeconomic diversity (we will use the Territorial Socio-Economic Index (IST) to determine socioeconomic conditions. This is a synthetic index that summarizes in a single value several socioeconomic characteristics of the population by small areas. The index includes data on employment, educational level, immigration and income of all people living in each territorial unit, based on six sectoral indicators [31]) and that can be used by health professionals working with young people.
- To provide a new framework for the WSJ that is competitive and effective with regard to the affective-sexual education of young people.
- To involve the target population in the creation of this intervention for their emotional and sexual wellbeing.

#### 1.2.3. Qualitative Part of the Study

Objective: To analyse experiences and opinions on the WSJ of people aged 14 to 25 from a social constructivist perspective (is a theoretical and methodological approach that supports the collective generation of meanings through language and social interaction [32]). Participants will be of different sexes, gender identities, sexual orientations and socioeconomic conditions, from urban, semirural and rural areas of Catalonia. WSJ knowledge, use, usability, accessibility (digital equity-is the fair distribution of digital resources throughout the entire population [33]-), usefulness and relevance of content will be assessed in order to improve and adapt its resources.

#### 1.2.4. Quantitative Part of the Study

General objective: To understand how the youth population of Catalonia evaluates the WSJ.

Specific objectives:

- To determine what content of the WSJ receives the best rating by young people.
- To identify whether assessment of content and usability of the WSJ varies according to the following factors: age, sex, gender identity, sexual orientation, culture of origin, socioeconomic status, area of residence (urban, semiurban or rural) and motivation for consulting the website (of their own accord or suggested by a professional).
- To identify whether assessment of content of the WSJ is different in young people that had previously used it.
- To assess if having previously consulted the ASSIR unit is associated with a different assessment of the WSJ content.
- To determine if the target population knows about the WSJ, and what percentage use it and consider it useful.
- To evaluate if socioeconomic conditions determine the use of paper vs. digital questionnaires.
- To determine if young people in Catalonia visit other affective-sex education websites, and which are the most popular.
- To determine if young people in Catalonia follow any influencers for affective-sexual education, and which influencers are the most popular.
- To determine the percentage of young people in Catalonia that visit pornography websites.

## 2. Materials and Methods

### 2.1. Design

We will use a parallel convergent triangulation design [32] and concomitantly implement a qualitative and quantitative phase. When these phases finish, we will prepare a single report triangulating the results of the two phases. Approval dates for this protocol were February 2017 and July 2020 for the qualitative part and entire mixed-methods part of the project, respectively. The study will last three years and participants will be young people living in Catalonia.

#### 2.1.1. Qualitative Part of the Study

The study will adopt an explanatory/interpretative model within a social constructivist framework. It will aim to describe and interpret social meanings generated collectively, i.e., the experiences and opinions of young people regarding the WSJ, affective-sexual education and their experience and needs regarding their sexuality. Investigators will have to discriminate between the hegemonic and counter-hegemonic discourses that originate from these social constructs [32,34].

#### 2.1.2. Quantitative Part of the Study

This is an observational, descriptive and cross-sectional quantitative study, for which an “ad hoc” questionnaire has been created.

### 2.2. Researchers’ Description

Since this protocol uses mixed methods, how the researchers’ background might influence the analysis must be explained. The principal investigator and most researchers are midwives working at ASSIR units, where they offer affective-sexual education to young people at individual consultations as well as in the community, at education centres and various associations and public spaces.

### 2.3. Sample/Participants

Participants will be teenagers and young adults aged 14 to 25 able to express themselves in one of the two official languages of Catalonia (Catalan or Spanish). We will exclude people who do not wish to participate in the investigation and people under 16 who wish to participate but whose parents/legal guardians do not sign the informed consent form. 

#### 2.3.1. Qualitative Part of the Study

The sampling will be theoretical. Participants will have to meet different profiles defined by representativeness criteria specified by the research team in order to provide heterogeneity to the sample and until reaching theoretical saturation of technical data. The representativeness criteria are age (two groups: 14–18 and 19–25), rural and city areas, different socioeconomic conditions, all sexes (male/female/intersex), different gender identities (male, female, transgender, non-binary, fluid and other) and different sexual orientations (heterosexual, homosexual, plurisexual, asexual).

#### 2.3.2. Quantitative Part of the Study

We have devised three methods for participant recruitment: (1) convenience sampling will be used in education centres (high schools, vocational training schools, training and insertion programs and universities); (2) consecutive sampling will be used to recruit young people who consult the ASSIR units; (3) young people who access the WSJ of their own accord will be invited to participate. These three methods will be used to invite participants to join the study; participation will be voluntary. Recruitment is more fully developed in the data collection Section 2.4.2.

Based on the size of the effect of the differences in the assessment of the WSJ contents, and depending on whether they consulted the website of their own accord or not, a total sample of 776 participants will be required, accepting an alpha risk of 0.05 and a beta risk of 0.2 in a two-sided test. Up to 388 subjects will be necessary in the first group and 388 in the second to find a statistically significant proportion difference, expected to be of 0.5 in group 1 and 0.4 in group 2.

### 2.4. Data Collection

#### 2.4.1. Qualitative Part of the Study

Focus groups and in-depth interviews will be used to collect information. A focus group consists of a group interview led by a moderator using a topic/interview script (the script of this project is available in Supplementary Material Table S1), with the additional participation of an observer [35]. Focus groups are based on the concept that group interaction facilitates reflection on individual and shared perspectives. They last between 90 and 120 min and include 4 to 12 people [36]. Focus groups will be audio- and video-recorded, and the recording will be stored in an encrypted file that prevents participant identification. The recordings obtained will be confidential, accessible only by informants and the research team and eliminated after one year. We have planned to conduct 10 focus groups, although this number might change based on the sufficiency and relevance of the information obtained, until saturation is achieved [32]. In-depth individual interviews will be used when participants feel they need more confidentiality and in cases of difficulty recruiting enough young people with specific profiles.

In the context of COVID-19, we might need to conduct virtual focus groups and/or individual interviews using the Microsoft Teams platform.

The recruitment of participants will take place at the ASSIR units during individual consultations, Youth Afternoons, the educational centres collaborating on the study and various youth associations, including those advocating for affective-sexual and gender diversity rights across all of Catalonia. Researchers will select potential informants and explain that they will receive a call from the principal investigator of the project, inviting them to participate in a group interview.

#### 2.4.2. Quantitative Part of the Study

In the context of COVID-19, we have both an online (Microsoft Forms) and paper version of the questionnaire (Teleform). The online questionnaire can be accessed via direct link or QR code. The questionnaire consists of 24 closed questions and 2 open-ended questions. The questionaire is available in Supplementary Material (Figure S1).

- At the education centres: The recruiting researchers will communicate with these centres to disseminate the questionnaire among students. The centre directors will decide if their centre will use the online or paper questionnaire.
- At the ASSIR units: a wide network of professionals are also researchers that will recruit for the study. The study will be disseminated through posters in the waiting rooms and at the counter of each ASSIR unit. These posters will contain the study instructions and the QR code for the questionnaire so that young people can quickly download it to their mobile phone and answer it. There will also be a paper version of the questionnaire for people unable to access it digitally. Digital users of the ASSIR will be sent the link to the questionnaire with information to encourage them to answer on the same platform they used for the consultation (email, WhatsApp^®^, e-consultation).
- WSJ users who make email inquiries will automatically receive an email with the direct link to the questionnaire, encouraging them to participate after explaining that this is a study to evaluate the website. The study will be also advertised on the WSJ homepage through images with a direct link to the questionnaire.

Whatever the origin of the participant, the variables that will be evaluated for all the participants in the sample through the questionaire are the following:

Sociodemographic variables: age, sex, gender identity, sexual orientation, country/culture of origin, years of residence in Spain, postal code, socioeconomic condition of the postal code according to the Territorial Socio-Economic Index, city/rural/semirural residence, current job, ongoing studies, type of access to the questionnaire (electronic or paper).

Variables related to the WSJ: prior knowledge of the WSJ, use of the WSJ, usability of the WSJ, usefulness of the WSJ, rating of affective-sexual education content.

Variables related to social media: ability to access to digital resources, use of other webpages of affective-sexual education, following affective-sexual education influencers, use of pornography webpages.

Variables related to study recruitment: Participants from educational centers and ASSIR units will access the survey by indication of the professionals. The third contacting group will access it by themselves, without anyone’s indication. The sample calculation has been performed based on this variable.

### 2.5. Ethical Considerations

The focus groups will be audio- and video-recorded. The individual face-to-face interviews will only be audio-recorded. The transfer of voice and image rights in the case of focus groups and of voice rights only in the case of individual interviews will be requested. In the context of COVID-19 (data collection for this study coincided with the COVID-19 pandemic during times of significant restrictions in Catalonia, Spain, such that the measures explained in this section had to be taken to continue implementing the project. These changes were accepted by the Research Ethics Committee.), we might audio- and video-record virtual interviews using Microsoft Teams. Participants must give their informed written consent. Participants under the age of 16 must also present the consent of their parent or legal guardian. This is known as double consent. Participants aged 16 to 18 are considered to be mature minors and will not need the consent of their parent or legal guardian. In the quantitative study, the questionnaires are anonymous and voluntary. Participants under the age of 16 must provide an informed consent form signed by them and their parent or legal guardian.

This protocol follows the tenets of the Declaration of Helsinki [37]. The research protocol was approved by the Research Ethics Committee (REC) of the Institut d’Investigació en Atenció Primària Jordi Gol (IDIAP). Approval dates were: February 2017 (Ethics reference number: P17/048) for the qualitative part of the study; July 2020 (Ethics reference number: 19/074-P) for the whole mixed-methods project. It also has the approval of the Management of ASSIR Catalunya and the editorial committee of the WSJ. All the directors of the education centres where the study will be conducted will be adequately informed.

Due to COVID-19, Microsoft Forms and Microsoft Teams software might be used. In this case, we will guarantee data protection, in accordance with the Regulation (EU) 2016/679 of the European Parliament and Council of April 27 on Data Protection (RGPD) and the Spanish Organic Law 3/2018 of December 5, on the Protection of Personal Data and guarantee of digital rights. The online questionnaire can only be answered by people aged 16 and over since the necessary consent cannot be obtained from children under 16. Consequently, the first question in the online questionnaire is if they agree to answer it, and the second if they are 16 or older. Responses from those under 16 will be collected using the paper questionnaire at the education centres and ASSIR units.

### 2.6. Data Analysis

#### 2.6.1. Qualitative Part of the Study

A discourse analysis (Why do they say this?) of participants in the focus groups and individual interviews will be conducted [38,39]. We selected discourse analysis because we aim to understand the hegemonic discourses and counter-discourses constructed by young people about the affective-sexual education they receive [40]. For this analysis, we will use ATLAS- ti (version 8).

#### 2.6.2. Quantitative Part of the Study

We will perform a descriptive analysis of all variables, using frequencies and percentages for categorical variables and mean and standard deviation for age and years living in Spain. Familiarity with the website will be summarized with a percentage and confidence interval. The bivariant analysis will be performed using Pearson’s chi-squared test or Fisher’s exact test to find associations between the assessment of WSJ and the sociodemographic variables. If the bivariant analysis shows any associations, a multivariant analysis using a regression model with assessment of WSJ as the outcome variable is planned. The anlaysis will be performed disaggregating by gender. Significance level is set at 5%. SPSS statistical package for Windows, version 25.0, will be used for all analyses.

### 2.7. Rigour

We will apply the quality and rigour criteria proposed by Guba and Lincoln [41], i.e., credibility, transferability, consistency and confirmability. Triangulation will be used for collecting information and for analysis. At the end of each focus group, the interviewer will briefly summarize the topics discussed and outline the contribution of each person, so that participants have the opportunity to make changes. Additionally, all participants will receive their transcripts for validation.

## 3. Discussion

This study should reveal the perceptions, needs and deficiencies regarding sex education of young people and help understand how they live, think and express their sexuality.

Authors on affective-sexual health agree that sexual health programs are not effective without the experience and perspective of young people [8,9,42].

### Limitations

Often, young people find it difficult to make commitments, and thus effective recruitment becomes more complex. Double consent, required of people under the age of 16, demands more resources and further hinders participation. Despite these limitations and in agreement with the literature, the proposed methodology is considered adequate.

## 4. Conclusions

The evaluation of the WSJ as a public health resource requires both (1) affective-sexual and gender diversity and (2) a socioeconomic perspective. The results of this study should provide the basis on which to build a new, effective model of affective-sexual education for young people.

## Data Availability

The data that support the findings of this study are available on request from the corresponding author. The data are not publicly available due to privacy or ethical restrictions.

## Supplementary Materials

The following supporting information can be downloaded at: https://www.mdpi.com/article/10.3390/ijerph192416586/s1, Figure S1. Questionnaire. Table S1. Script for focus groups and in-depth interviews.
