# Supplementary material for: Adolescents and Young Adults Evaluating a Website for Affective-Sexual Information and Education: A Mixed-Methods Study Protocol
Source: Int J Environ Res Public Health. 2022 Dec 9;19(24):16586. doi: 10.3390/ijerph192416586 (PMC9778971; doi:10.3390/ijerph192416586)
Supplement: Supplementary file 1 [file ijerph-19-16586-s001.zip › Figure S1_Questionnaire_ijerph- 2045991.pdf]

Figure S1. Questionnaire

1. Where did you get this questionnaire?
   - An educational centre
   - A health centre
   - Other
2. Year of birth

3. Sex
   - Penis
   - Vulva
   - Intersex
4. Sexual orientation
   - Heterosexual
   - Bisexual
   - Homosexual
   - Other
5. Gender identity
   - Man
   - Woman
   - Non-binary
   - Other
6. Country of origin of your parents/legal guardians (if they are from different countries, write both and indicate who is from where. E.g.: Colombia: mother; Argentina: father)

7. How many years have you lived in Spain?

8. City/town where you live

9. Postal code

10. Ongoing education
    - I don't study
    - Secondary school
    - High school
    - Intermediate level vocational training
    - Higher level vocational training
    - Training and insertion programs
    - University

11. Do you have a job?
    - Yes
    - No

12. Are you familiar with the website Sexe Joves?
    - Yes
    - No

13. Do you think the young people in your milieu know of it?
    - Yes
    - No

14. Do you use it?
    - Yes
    - No

15. Only answer if you do use it. From what device do you use it?
    - Mobile phone
    - Tablet
    - Computer

16. Only answer if you do use it. Is it easy to access the content you are looking for?
    - Yes
    - No

17. Only answer if you do use it. Do you find it useful?
    - Yes
    - No

18. Only answer if you do use it. What do you use most? (You may tick more than one option.)
    - Content
    - Chat
    - Email

19. Do you visit other websites about affective-sexual education?
    - Yes
    - No

20. Which ones?

21. Do you follow any influencers who talk about affective-sexual education?
    - Yes
    - No

22. Could you tell us their names?

23. Have you ever visited porn websites?
- Yes
- No

24. Rank the following content in order of importance to you.

	Unimportant	Important	Indispensable
Emotion and sexuality			
Knowledge of your body			
Petting			
The first time			
Contraception			
Emergency contraception			
Pregnancy			
Abortion			
Sexually transmitted infections			
Abuse, harassment and sexual violence			
Sex and drugs			
Virtual sex			
Cyberbullying			
