# Supplementary material for: Adolescents and Young Adults Evaluating a Website for Affective-Sexual Information and Education: A Mixed-Methods Study Protocol
Source: Int J Environ Res Public Health. 2022 Dec 9;19(24):16586. doi: 10.3390/ijerph192416586 (PMC9778971; doi:10.3390/ijerph192416586)
Supplement: Supplementary file 1 [file ijerph-19-16586-s001.zip › Table S1_ijerph- 2045991.pdf]

Table S1. Script for focus groups and in-depth interviews.

Topics of Interest	Guiding Questions
Personal information	Age, population, ongoing education, profession, current employment, etc. (Questions about sex, gender identity, sexual orientation, country of origin of parents/legal guardians, number of years residing in Spain, if they have a partner, children, number and age of children, etc. will be asked in the days before the focus group—on an individual and confidential basis—when the principal investigator contacts each participant)<br>Belonging to a group/community.<br>Responsibility within this group/community.
Familiarity with the WSJ	Are you familiar with the website Sexe Joves? How did you learn about it? Do you think young people are familiar with it?<br>Are the young people specifically in your milieu familiar with it?
Use of the WSJ	Do you use it? Do you think young people use it? Do you know people in your milieu who use it? Do you think young people use the content available on it? Do they use the chat on it?
Accessibility (Digital equity)	Can you easily access the WSJ? You have enough mobile data to access it any day of the month? Do you have Wi-Fi at home so that you can access it? Do you think most young people can access the WSJ any day of the month? What about the young people specifically in your milieu?<br>Do you think that affective-sexual education imparted on websites reaches all young people? Does it reach those in your milieu? What about those who do not have mobile data? How do you think affective-sexual education should be imparted so that it reaches all young people?
Usability of the WSJ	Do you find it easy to use? Is the written language on the website understandable? Do you find what you're looking for quickly? Is there too much written language? Are the images motivating? Do they feel close to you? Do you think it meets your information and education needs regarding sexuality?<br>Are the images representative of all young people? Are they representative of all affective-sexual and gender diversity? Are they representative of the full diversity of bodies, racialized people, etc.? Can all young people see themselves identified in these images?
Utility of the WSJ	Do you find it useful? Do you think it is a valid and suitable channel for your affective-sexual education?
Content of the WSJ	Do you find the content on the website interesting? Would you add other content? Would you remove content?<br>Do you think the content is reliable? Would you trust it?<br>Do you think the WSJ responds to the questions about sexuality that all young people might have, regardless of their sex, gender identity or sexual orientation? And regardless of their socio-economic status?
Affective-sexual education	Have you received affective-sexual education?<br>Where? From your family? At school? From health professionals? Which professionals, specifically? Do you remember about which topics? Do you feel you have received quality affective-sexual education? Did you receive it when you needed it or too late?<br>Do you think that the lack of affective-sexual education causes young people to search for answers in pornography?<br>If you did not receive enough affective-sexual education, do you think that this generated, or still generates, fears, insecurities, difficulties, etc. related to sexuality?<br>What topics related to sexuality do you think you haven't received any information or education about?<br>When do you think you should start receiving affective-sexual education? Where should it take place in order to reach the entire population?

Do you believe that the WSJ can become a good means of support for a comprehensive, specific and cross-cutting affective-sexual education for the entire population?
